# Uncovering the effect of waterlogging stress on plant microbiome and disease development: current knowledge and future perspectives

**DOI:** 10.3389/fpls.2024.1407789

**Published:** 2024-06-06

**Authors:** Anshika Tyagi, Sajad Ali, Rakeeb Ahmad Mir, Sandhya Sharma, Kumari Arpita, Mohammed A. Almalki, Zahoor Ahmad Mir

**Affiliations:** ^1^ Department of Biotechnology, Yeungnam University, Gyeongsan, Republic of Korea; ^2^ Department of Biotechnology, School of Life Sciences, Central University of Kashmir, Ganderbal, Jammu and Kashmir, India; ^3^ ICAR-National Institute for Plant Biotechnology, New Delhi, India; ^4^ Department of Biological Sciences, College of Science, King Faisal University, Al-Ahsa, Saudi Arabia; ^5^ Department of Plant Science and Agriculture, University of Manitoba, Winnipeg, MB, Canada

**Keywords:** waterlogging, plant microbiome, hypoxia, pathogens, signaling, disease

## Abstract

Waterlogging is a constant threat to crop productivity and ecological biodiversity. Plants face multiple challenges during waterlogging stress like metabolic reprogramming, hypoxia, nutritional depletion, reduction in gaseous exchange, pH modifications, microbiome alterations and disease promotion all of which threaten plants survival. Due to global warming and climatic change, the occurrence, frequency and severity of flooding has dramatically increased posing a severe threat to food security. Thus, developing innovative crop management technologies is critical for ensuring food security under changing climatic conditions. At present, the top priority among scientists is to find nature-based solutions to tackle abiotic or biotic stressors in sustainable agriculture in order to reduce climate change hazards to the environment. In this regard, utilizing plant beneficial microbiome is one of the viable nature based remedial tool for mitigating abiotic stressors like waterlogging. Beneficial microbiota provides plants multifaceted benefits which improves their growth and stress resilience. Plants recruit unique microbial communities to shield themselves against the deleterious effects of biotic and abiotic stress. In comparison to other stressors, there has been limited studies on how waterlogging stress affects plant microbiome structure and their functional traits. Therefore, it is important to understand and explore how waterlogging alters plant microbiome structure and its implications on plant survival. Here, we discussed the effect of waterlogging stress in plants and its microbiome. We also highlighted how waterlogging stress promotes pathogen occurrence and disease development in plants. Finally, we highlight the knowledge gaps and areas for future research directions on unwiring how waterlogging affects plant microbiome and its functional traits. This will pave the way for identifying resilient microbiota that can be engineered to promote their positive interactions with plants during waterlogging stress.

## Introduction

In recent decades, harsh environmental conditions, such as floods, drought, and extreme temperatures, have caused a significant drop in agricultural yields across the globe (Kreibich et al., 2022; Furtak and Wolińska, 2023). According to the Food and Agriculture Organization of the United Nations (FAO), an increase in food production of around 70% by 2050 is necessary to fulfill the demand of an expanding population (FAO, 2021). However, in fulfilling this demand, there is need to develop future climate resilient smart crops in sustainable agriculture. Numerous problems arise in the forecasts of what the future holds for our global community, including how to strengthen food production in light of the escalating effects of climate change and rising population. Climate change has dramatically increased the magnitude and occurrence of environmental stressors like floods which affects crop productivity and food security (Kreibich et al., 2022). Among environmental stressors, waterlogging stress has emerged as a significant threat to agricultural output because it alters not only key plant physiological and biochemical features but also alters microbiome and soil physiochemical properties (Francioli et al., 2021; Nio and Mantilen, 2023). In nature, seasonal flooding is a regular occurrence in various ecosystems and has a favorable impact on biodiversity and production. Flooding may benefit agriculture by reloading soil nutrients in floodplains, creating new homes for wildlife, and reviving wetlands (Tonkin et al., 2018). However, unanticipated and uncontrolled floods, on the other hand, are one of the most damaging natural catastrophes, with the ability to wreak huge damage not only to agriculture and but also endangers public health (Kron, 2005). Climate models indicate that flooding events may become more frequent and severe in the near future (Jongman et al, 2014). Under flooding conditions, plants can be either completely submerged or partially submerged which can have distinct impact on their physiological, biochemical and morphological traits (Sasidharan et al., 2017). In other words, flooding is classified into two types: waterlogging, which occurs when water is on the soil surface and only plant roots are submerged and submergence, in which the whole plant can be either underwater/fully immersed or partially submerged (Jia et al., 2022). In the field, waterlogging can occur quickly after a heavy rainstorm or as a result of flood, which leads plants to hypoxic conditions (Voesenek et al., 2016).

Plants under waterlogging stress becomes more susceptible to microbial pathogens which further endangers their survival (Moslemi et al., 2018). On the other hand, waterlogging also leads dramatic alteration in root microbiome which has huge impact on plants survival under unfavorable conditions (Francioli et al., 2022; Leelastwattanagul et al., 2023). In nature, plants are associated with diverse and taxonomically structured microbial communities including bacteria, fungi, and viruses, which are called the plant microbiota (Trivedi et al, 2020). There are numerous reports which highlight the importance of beneficial microbes in improving not only plant growth but also their tolerance to different stressors (Bokhari et al, 2019; Shekhawat et al, 2021; Timmusk et al., 2023). Some of the key function’s microbes can assist plants are nutrient availability, modulation of growth and defense phytohormonal signaling cascades, enhances stress tolerance and soil fertility (Ali et al., 2022a, Ali et al, 2022b, Ali et al., 2023). Despite significant progress in crop cultivar genetic modification and cultivation practices that reduce waterlogging effects, the impact of rhizosphere microorganisms in plant resistance to waterlogging has received little attention. To reduce flooding stress, two primary tactics may be implemented: traditional water management facilities (e.g., drainage, dikes) and natural based solutions (Zölch et al., 2017). Also, agronomic solutions for dealing with submergence or waterlogging include creating standard models for predicting and assessing crop loss due to floods for risk management, decision making and economic insurance. Exploring plant microbiome under waterlogging stress can provide novel nature-based strategy for improving plant tolerance to waterlogging stress. Previous studies have reported the microbial inoculation can ease waterlogging induced effects in plants. For example, inoculation of plants with Bacillus sps, producing 1-aminocyclopropane-1-carboxylic acid deaminase, lower stress-induced ethylene levels, thereby protecting plants from waterlogging stress (Ali and Kim, 2018). Similarly, Farwell et al. (2007), revealed that inoculation of *Pseudomonas putida* UW4 generates ACC deaminase, which mitigates the effects of waterlogging and metal stress. Several ACC deaminase-producing bacterial strains, including *Serratia ureilytica*, *Achromobacter xylosoxidans*, *Ochrobactrum rhizosphaerae*, and *Herbaspirillum seropedicae*, were isolated from the rhizosphere of waterlogged *Ocimum sanctum* which may protect plants from waterlogging-induced damage (Compant et al., 2019). These studies further support the notion that microbiota can be an important tool for mitigating waterlogging stress in sustainable agriculture. There are many reports which have shown that microbiome as a significant component for improving plant health and resilience to environmental stressors (Koskella et al., 2017; Lau et al., 2017; Compant et al., 2019). Unlike other stresses, there have been few studies examining the influence of waterlogging on plant microbiota. There are reports that waterlogging promotes anaerobes and disease-causing pathogens which jeopardize plants survival (Hsu and Shih, 2013; Leelastwattanagul et al., 2023). However, it is likely that plants may also recruit stress-relieving microbiome, with the ability to promote or adapt to waterlogging stress necessitates future investigations. The effects of waterlogging stress on microbial diversity and plant microbiome interactions in not fully explored despite the availability of high throughput tools. This mini review offers an update on how waterlogging stress affects plants and their microbiota. First, we discuss the effect of waterlogging in plants and signaling evolved. Next, we discussed the effect of waterlogging stress on plant microbiota. We also highlight how waterlogging promotes pathogen distribution, distribution and disease severity in different plant systems.

## Waterlogging stress in plants

Waterlogging affects multifaceted morphological, physiological and biochemical traits in plants which are crucial for their growth and survival. The main challenges plants face during waterlogging stress are reduction in the rate of gas exchange, hypoxia, low nutrient absorption, preventing aerobic respiration, increased reactive oxygen species (ROS) and ethylene levels (Ashraf, 2012; Tamang et al., 2014). Waterlogging also affects root system architecture such as growth inhibition of lateral roots which is due to the interference of ethylene with local auxin signaling (Shukla et al., 2019). Waterlogging altered root system fails to transport water and nutrients to aerial parts thereby causes reduced apoplastic water movement (Sauter, 2013). Waterlogging stress also affects chemical or hydraulic signals that cause stomatal closure, eventually contributing to reduced leaf development (Li et al., 2015). Another common response to floods is a reduction in photosynthesis. On the other hand, waterlogging triggers the accumulation of toxic compounds, carbon starvation and cytoplasmic acidification which eventually leads to plant death. On the other hand, plants vary in their capacity to survive the detrimental effects of waterlogging due to their rapid or induced modifications in plant traits, like plant height, adventitious roots aerenchyma production, changes in leaf anatomy, improved shoot elongation, starch storage hyponasty, barriers against radial oxygen loss (Voesenek et al., 2016). For example, taller plants, in particular, with aerenchyma content and larger specific leaf area may retain greater levels of gas exchange during a flood and so continue to grow (Colmer et al., 2019). Furthermore, certain plants may store enormous amounts of starch in their underground structures, alter their metabolic rates, and have the ability to develop quickly after the flood waters subside (Voesenek and Bailey-Serres, 2015).

Plants under go rapid metabolic and anatomical reprogramming during waterlogging stress in order to survive (Tyagi et al., 2023). Previous research has shown that waterlogging triggers local hypoxia-driven responses in the roots as well as systemic responses in the shoots, including changes in hormonal dynamics, metabolic reprogramming, ubiquitin-dependent protein degradation, and a variety of other molecular and metabolic responses (Hsu et al., 2011). Two hormones namely Abscisic acid (ABA) and ethylene (ET) were identified as key drivers for systemic signaling during water stress (Hsu et al., 2011; TSAI et al., 2014). One of the earliest responses to anoxia conditions in plant roots or shoots is the activation of calcium and reactive oxygen species (ROS) signaling cascades and suppression of mitochondrial respiration (Yang et al., 2023). Some of the key players like vacuolar H^+^/calcium transporter CATION/PROTON EXCHANGER 1 (CAX1) and the RESPIRATORY BURST OXIDASE HOMOLOGs D and F (*RBOHD* and *RBOHF*) that drive early response during waterlogging have been identified that regulates distinct anoxia response like aerenchyma formation (Liu et al., 2017; Yang et al., 2022). Recently, Peláez-Vico et al. (2023), identified GLUTAMATE-LIKE RECEPTOR 3.3 and 3.6 (GLR3.3 and GLR3.6) calcium channels, RBOHD, and aquaporin PLASMA MEMBRANE INTRINSIC PROTEIN 2,1 (PIP2,1) proteins as potential players involved in waterlogging systemic signaling in Arabidopsis. These studies provide novel insights on how plants respond to waterlogging stress at molecular level. However, future studies are required to unravel early, localized and systemic signaling form root to shoot and also the role of cell wall sensors and calcium channels, hormones and transporters during waterlogging stress, as well as how they regulate signal perception and transduction.

## Plant microbe interactions under stress conditions

Plant-microbiome interactions are complex which can be beneficial or harmful in nature. For example, beneficial microbiome provides an array of benefits to plants such as nutrient availability and uptake, nitrogen fixation, promote growth, antagonism towards pathogens, boost stress resilience and improve soil fertility (Ali et al., 2023). In contrast, harmful microbiota can be saprophytic, biotrophic, hemi biotrophic and necrotrophic inhibiting or killing the host through a variety of mechanisms (Wille et al., 2019). There have been numerous studies highlighting the plant-microbe interaction mechanisms, such as how plants respond to microbial colonization and how microbial diseases and symbionts modify plant cellular processes (Cheng et al., 2021). Interestingly, plant microbiome is an essential determinant of plant health and also one of the important drivers for plant survival under stressful conditions (Compant et al., 2019). Plant beneficial microbiomes or their metabolites are often used bioinoculants or biostimulants to enhance sustainable plant development, and have emerged as a viable alternative to agrochemicals that have negative environmental and health consequences.

Plant microbiome interactions occurs at different plant compartments with distinct habitats like phyllosphere, endosphere and rhizosphere. Plant microbiome assembly is an intricate process which is highly influenced by numerous genetic, biochemical, and physiological and environmental factors. For example, microbes have different growth conditions in terms of physiology, nutrients, pH, temperature and moisture in addition to host factors all of which can have significant impact on their assembly and host interactions in above and below ground plant organ systems (Trivedi et al., 2020). Plants ability to produce diverse chemical compounds like hormones, flavonoids mucilage, and other chemicals from roots affects microbial development, attracts particular bacteria, and can vary the rhizosphere features. However, soil microbiota, on the other hand, are sensitive to environmental changes, which has significant impact on plant survival (Zhou et al., 2023). Increased climate change and other harsh environmental stressors have not only direct effect on plant growth and yield production but also on its beneficial microbiome and their interactions. As, climate change is increasing the frequency and intensity of drought, flooding, and global temperatures is rising all of which changes the composition and activity of plant microbiomes, potentially affecting host functional attributes. Many studies have shown that environmental stressors change plant microbiome which can have distinct impact on growth and adaptive traits or can be either beneficial or detrimental to their host plants. For example, under drought stress plant recruit selective drought tolerant bacterial taxa which supports their growth under drought stress (Fitzpatrick et al., 2020). Similarly, under salinity stress plant shape unique microbiome which alleviates their salt induced effects (Xu et al., 2020). Similarly, endophytes have been found to promote seed germination during heat and drought stress (Hubbard et al., 2019). According to Wipf et al. (2021), *Sorghum bicolor* under drought and heat stress shapes particular microbiota belongs to Actinobacteria which are known to promote growth under stress conditions. On the other hand, environmental cues can have detrimental impact on plants by promoting harmful microbiota. For example, during waterlogging anaerobes and pathogens can dominate which can have detrimental impact on plant growth and survival (Moslemi et al., 2018). Previous research has demonstrated that flooding affects the root microbiome by decreasing immunological modulator beneficial bacterial communities making plants more susceptible to disease (Soltani et al., 2010; Kavamura et al., 2021). Climate change can modify pathogen abundance and behavior, disrupt host-pathogen interactions, and stimulate the formation of novel diseases (Cohen and Leach, 2020).

## Effect of waterlogging on plant microbiome

Microbes associated with root system have a significant influence on the soil environment, regulating numerous soil biochemical processes as well as plant growth and adaptive (Sun et al., 2024). Like other stressors, flooding has a direct influence on soil and root microbiome by gradually depleting O2 in soil pores which are filled with water. The shift from oxygenated to anoxic soil affects the microbial makeup from a preponderance of aerobic organisms, to a higher presence of facultative anaerobes, and eventually to the dominance of strict anaerobes. Flooding alters microbial communities in bulk and rhizo-sphere soils (Lin et al., 2011; Hamonts et al., 2013; Francioli et al., 2021). Because the bulk soil is the primary source of microorganisms recruited by plant roots in the rhizosphere (Bulgarelli et al., 2012; Bonito et al., 2014), flooding’s impact on the microbial composition of the bulk soil can likewise influence the microbiome of the rhizosphere. Several research on rice plants have shown how flooding impacts the microbiome, mostly in terms of bacteria, although archaea, oomycetes, fungus, and viruses remain largely unknown. Flooding has been demonstrated to change rhizospheric and bulk soil microbial populations (Iniesta-Pallarés et al., 2023). Previous research has highlighted the impact of flooding on the rice phyllosphere microbiome, with Firmicutes (54%) and Bacillus (52.63%) being the leading species in flooded rice plants. According to Tian et al. (2015), the amount and duration of floods reduce plant microbial endophyte colonization. Under normal conditions, the microbiome profiling showed that the presence of beneficial microbial communities such as *Desul-fitobacterium*), a nitrogen and carbon dioxide-fixing bacteria *Amnibacterium kyonggiense*, phosphatase and beta-glucosidase-producing bacteria, Streptomyces and Chaetomium pathogen inhibiting, and plant growth hormone-producing microbes like *Trichoderma*, *Talaromyces Promicromonospora* and Penicillium (Hyakumachi, 1994; Salas-Marina et al., 2011). However, soil microbiome profiling in sugarcane during waterlogging showed the dominance of plant detrimental microbial communities like pathogens and growth-inhibiting bacteria (Leelastwattanagul et al., 2023). Waterlogging also effects plant mycobiome in sugarcane by increasing Basidiomycota and reducing Ascomycota which contains many plant pgroth promoting fungal genera like *Trichoderma, Aspergillus, Talaromyces, Exophiala*, *Cladosporium, Phoma, Penicillium, Purpureocillium, Chaetomium*, and *Phomopsis* (Leelastwattanagul et al., 2023). Similarly, *Myricaria laxiflora*, a riparian shrub that frequently encounters periodic summer floods, has decreased endophyte diversity in anaerobic conditions. A recent study on spring wheat (*Triticum aestivum*) found that flooding stress causes substantial alterations in the makeup of the rhizosphere microbiome (Francioli et al., 2022). They found that anaerobic bacteria belonging to phyla Desulfobacterota and Firmicutes along with plant detrimental microbial taxa Geobacter and Clostridium were dominant than plant-beneficial bacterial taxa like *Sphingomonas* and *Streptomyces* which will have huge outcome on plant fitness and survival. There have been numerous studies on how flooding effected different microbial communities and their functional attributes. For instance, hypoxia triggered by flooding effects the plant mycorrhizal association mainly by inhibiting hyphal growth and AM spore germination (Tacon et al, 1983). Similarly, flooding also affects ecto-mycorrhiza (ECM) colonization and richness (Unger et al., 2009). Plants exposed to waterlogging stress reduced their ability to colonize with microbial endophytes as most of the endophytes colonizing terrestrial roots are obligate aerobes, and their survival is hindered under hypoxic conditions triggered by flooding (Li et al., 2010). Previous studies have reported that endophyte diversity was decreased in *Myricaria laxiflora* and rice plants during flooding stress (Tian et al., 2015). Flooding also alters phyllosphere microbiome structure in plants (Tian et al., 2015; Vishwanathan et al, 2020). For instance, rice culms exhibited a decrease in Gammaproteobacteria members in response to flooding stress, although Firmicutes members, particularly Bacillus species, appeared to adapt to flooding (Cui et al., 2019). In rice, flooding and heat stress dramatically changes root microbiome by enhancing the presence of bacterial alpha diversity and reducing the relative richness of Actinobacteria and Firmicutes which plays key role in carbon decomposition and soil fertility (Liu et al., 2023). In addition to root microbiome, flooding also effects leaf microbiome dynamics depending on the developmental stage, with younger plants experiencing a more dramatic disturbance in community formation (Francioli et al., 2022). Importantly, these studies reported that the change in microbiome composition was directly related to plant growth and development as well as adaptive responses.

**Figure 1:**
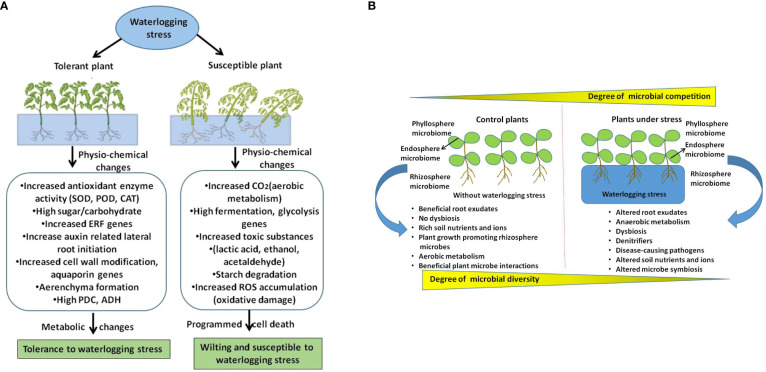
A schematic illustration shows how waterlogging affect plants and their microbiome. **(A)** Depicts waterlogging induced changes in tolerant and susceptible plants. **(B)** Shows how waterlogging stress affects microbiome structure by altering host driven factors and soil physicochemical properties which leads to dysbiosis and effects plant growth and adaptive traits.

In comparison with other stressors, how waterlogging changes plant root exudate chemistry that influence microbiome structure is not fully understood. It is well documented that plants undergo metabolic reprogramming from aerobic to anaerobic energy synthesis which can direct influence on root exudates. Therefore, it will be interesting to unravel the root exude diversity under waterlogging conditions in both model and crop plants which can provide novel insights on how plants influence its microbiome during waterlogging. However, the effect of waterlogging on soil physicochemical traits such as pH, structure, porosity, nutrients and oxygen reduction or reduced gaseous exchange are the primary factors have severe influence on microbial diversity and community activity (Neatrour et al., 2004; Yu et al., 2022). Further we have shown the effect of waterlogging on plant microbiome and its functional traits that are associated with plant growth and adaptive responses in **Figure 1**. In this schematic illustration we have highlighted host driven factors like metabolic shift from aerobic to anaerobic, altered root exudes, and soil based factors such as hypoxia, reduction in gaseous exchange, nutrition shortage that alter plant microbiome during waterlogging.

## Waterlogging increases pathogen distribution and disease severity in plants

Plants are constantly challenged by different microbial pathogens which causes significant yield losses (Ali et al., 2018; Mir et al., 2021). With climate change and occurrence of abiotic stressors has changed the distribution, host specificity and pathogenicity of microbial pathogens thereby posing serious threat to sustainable agriculture (Ali et al., 2023). Flooding not only affects plant growth but also makes them susceptible to number of pathogens. Flooding changes plants microbiome structure which have significant impact of their disease resistance. Indeed, flooding impacts the onset and development of various plant diseases by altering host vulnerability as well as the survival and pathogen distribution. In general, diseases and pests proliferate rapidly in high humidity circumstances due to enhanced germination and proliferation which ultimately leads to huge crop yield losses (Savary et al., 2019). Flooding promotes disease progression when plants get infected with oomycete or fungal diseases like *Phytopythium, Pythium, Phytophthora, Fusarium* (Wilcox, 1985; Moslemi et al., 2018). These pathogens cause more damage to waterlogged stress plants and leads to high mortality. During floods, increased root exudation of ethanol, carbohydrates, and amino acids can promote pathogen infection (Blaker and McDonald, 1981; Tyler, 2002). Flooding promotes disease development by altering beneficial microbial communities which are crucial for activating plant immune system. Previous studies have shown that flood affects immune modulator beneficial microbial communities belonging *Sphingomonas*, *Streptomyces, Flavobacterium, Saccharimonadia* and *Massilia* which leads to dis-ease progression (Soltani et al., 2010; Kavamura et al., 2021). Also, flooding promotes disease progression by affecting plant symbiotic association which are known to inhibit disease and pathogen distribution by their antagonistic or antibiotic potential, as well as activating systemic resistance induction. Francioli et al. (2021), reported an increase in *Clostridium* species in roots after floods which is commonly linked with root rot under waterlogged soils. Previous study has revealed that flooding in Ulmus minor plants changes root mycobiome and increases the development of root rot disease caused by *Plectosphaerella cucumerina* by altering the beneficial microbial communities (Martínez-Arias et al., 2020). Recent study has revealed that *Phytophthora medicaginis* a causative agent of root rot disease in chickpea, was more severe under waterlogging conditions (Dron et al., 2022). At present there are not effective control measures against Phytophthora root rot however, farmers are advised to avoid fields prone to waterlogging. Similar studies have demonstrated that waterlogging enhances the infection of phytopthora root rot in oak, avocado, and lucerne plants (Kuan and Erwin, 1980; Jacobs et al., 1997). In kiwi fruit, waterlogging triggers the severity of root rot disease caused by *Phytopythium vexans* and *Phytopythium chamaehyphon*, which causes more damage that waterlogging alone (Savian et al., 2020). Waterlogging has been shown to increase the prevalence of apple crown and root rot (Phytophthora spp.), banana vascular wilt (*F. oxysporum* f. sp. cubense), raspberry damping off (*Pythium irregulare*), and chili pepper verticillium wilt (*Verticillium dahlia*) (Aguilar et al., 2000; Sanogo et al., 2008; Li et al., 2015). On the other hand, waterlogging renders pigeon pea plants more vulnerable to fungal diseases including Fusarium wilt and Phytophthora blight, resulting in considerable output losses (Yohan et al., 2017). Previous study has reported that waterlogging increases the severity of disease in pea plants caused by *Mycosphaerella pinodes* and resulted in reduced root and shoot growth (McDonald and Dean, 1996). Above studies further provides the evidence that waterlogging enhances pathogen aggressiveness, their occurrence that causes more damage than waterlogging alone. Further we have summarized the case studies highlighting the effect of waterlogging on disease incidence and severity in different plants in (**Table 1**).

**Table 1:** Waterlogging induced pathogen occurrence and disease progression in different plants.

Plant	Waterlogging (WL)	Pathogen	Effect	References
Chickpea	WL	*Phytophthora* *medicaginis*	Enhanced disease progression and reduced the total plant biomass	(Dron et al., 2022)
Soybean	WL	*Pythium ultimum* *P. irregulare, P. aphanidermatum, and P. vexans*	Root discoloration. Reduced plant weight	(Kirkpatrick et al., 2006)
Wheat and barley	WL	*Fusarium poae*	Enhanced disease incidence and severity, Effects yield components and grain composition	(Martínez et al., 2019)
Bell Pepper	WL	*Phytophthora capsici*	Increased disease severity and plant mortality	(Bowers et al., 1990)
Apple	WL	*Phytophthora cambivora, Phytophthora cactorum* and *P. cryptogea*	Enhanced the severity of root rot disease	(Browne and Mircetich, 1988)
Soybean and tobacco	WL	*P. sojae and P. nicotianae*	Suppression of host disease resistance	(McDonald, 2002)
Kiwifruit	WL	*P. vexans and P. chamaehyphon*	Enhanced onset of disease and severity. High mortality rate	(Savian et al., 2020)
Potato	WL	*Erwinia carotovora*	Effect the expression of host defense genes and increased the severity soft rot.	(Rumeau et al., 1990)
Onion	WL	*Colletotrichum* sp.	Enhanced disease development	(Lopes et al., 2021)
Banana	WL	*F. oxysporum*	Increased the prevalence of wilt disease	(Aguilar et al., 2000)
Common bean	WL	*Pythium sps*	Enhanced disease development	(Li et al., 2015)
Pigeon pea	WL	*Fusarium and Phytophthora*	Increased disease incidence and development. Yield losses	(Yohan et al., 2017)
Peach	WL	*P. vexans and P. irregulare*	Enhanced disease severity	(Biesbrock and Hendrix, 1970)
Pea	WL	*Mycosphaerella pinodes*	Increased disease incidence Reduced root and shoot growth	(McDonald and Dean, 1996)
Alfalfa	WL	*Phytophthora megasperma*	Enhanced root damage and disease severity	(Kuan and Erwin, 1980)
Jarrah	WL	*Phytophthora cinnamomi*	Increased disease incidence and severity	(Burgess et al., 1999)

**Figure 2:**
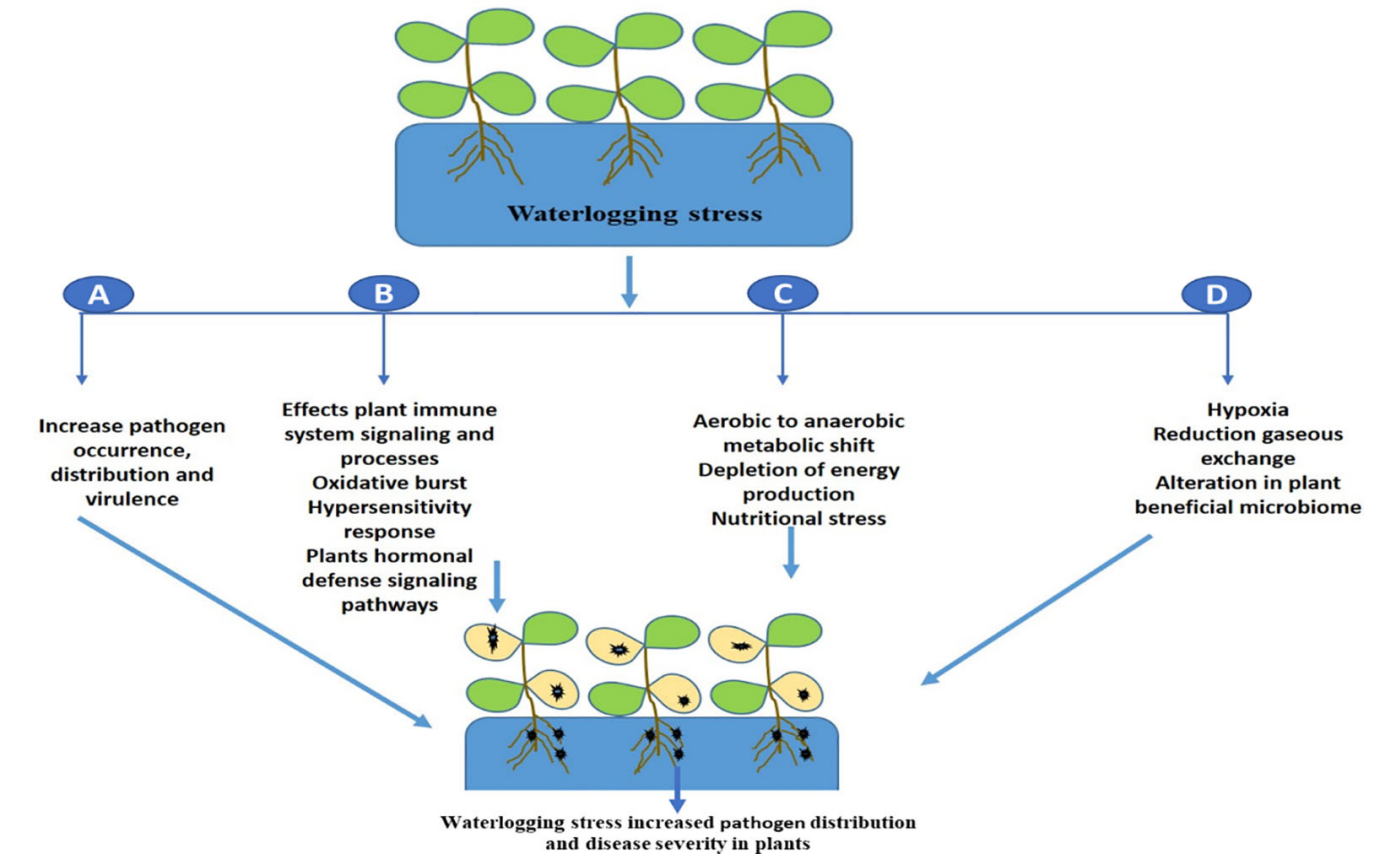
A schematic illustration shows how waterlogging affects different plant traits that promotes pathogen distribution and disease development. **(A)** waterlogging can enhance pathogen invasion or virulence, **(B)** it affects plant immunity signaling cascades which leads plant more vulnerable to diseases, **(C)** waterlogging induced metabolic shift, hypoxia, energy crisis and nutritional stress also promotes disease progression, **(D)** Alterations in beneficial microbiome or dysbiosis leads to pathogen dominance and disease progression.

The promotion of pathogenic microbes and disease progression in plants during waterlogging in mainly linked with energy deprivation (Moslemi et al., 2018), suppression of oxidative burst and the immune response, and hypersensitive cell death (McDonald, 2002). It will be interesting in future to decipher the molecular complexity of waterlogging and plant disease development and identify potential targets that suppresses plant immunity. Further we have shown the effect of waterlogging on disease development in plants in **Figure 2**. In contrast, hypoxia caused by floods can minimize disease development in plants by enhancing host defense responses as a result of the activation of a general stress response (Chung and Lee, 2020). Similarly, it was found that flooding stress lead the activation of plant immune signature transcriptional factors WRKY which modulated the expression of plant defense marker genes which have diverse antagonistic effect on microbial pathogens (Hsu et al., 2013). Future research is thus needed to determine how waterlogging affects plant immune signaling cascades utilizing different crop systems, since this would offer fresh perspectives on enhancing disease resistance.

## Conclusion and future perspectives

Waterlogging is a complex process which affects plant growth and its metabolic traits by reducing soil oxygen levels, soil nutrient utilization efficiency and altering microbiome. Over the last decade, there has been a great deal of interest in exploring the beneficial functional attributes of plant microbiomes, for crop improvement which have proven to be an effective nature-based solution to combat environmental stresses while safeguarding environmental and soil health. For instance, the development of drought or salinity tolerant microbiome synComs have been used by many researchers to improve the growth and adaptive responses in different plant systems under lab and field conditions. Therefore, there is need to utilize plant beneficial microbiome as a key strategy for mitigating waterlogging stress in plants and enhance crop productivity. This will require an in-depth study using different omics and synthetic biology approaches to decipher how plant microbiome responds and adapts to waterlogging stress. Also, harnessing genome editing tools and synthetic biology to engineer plants to produce root exudates that can shape unique stress resilient microbiome is another viable strategy for improving plant waterloging tolerance and growth traits. There is need to explore the microbiome of waterlogging tolerant crops or wild varieties which may lead to the identification unique stress resilient microbiota that might promote the plant growth and survival under waterlogging conditions. Future studies should also unwire how waterlogging driven metabolic shift in plants alter root exudate chemistry that will eventually shape distinct microbiome communities. In light of climate change, microbes are the best candidates to explore because of their rapid natural adaptability to environmental extremes and nourish under extreme growth conditions. At the same time, how microbiome dysbiosis occurs during waterlogging stress that triggers disease development or pathogen distribution warrants future investigation. Finally, we recomend for designing future flood modelling tools which will be an effective way for evaluating hypothesis and examining different situations, particularly with relation to the health of plants, microbial communities, plant pathogen interactions and soil health. This would require the collaboration of plant scientists from different backgrounds to design future flood modelling in order to prevent flood induced damage to sustainable agriculture.

## Author contributions

AT: Conceptualization, Methodology, Software, Validation, Formal analysis, Investigation, Resources, Data curation, Writing – original draft, Writing – review & editing, Visualization, Supervision, Project administration, Funding acquisition. SA: Conceptualization, Methodology, Software, Validation, Formal analysis, Investigation, Resources, Data curation, Writing – original draft, Writing – review & editing, Visualization, Supervision, Project administration, Funding acquisition. RAM: Methodology, Software, Validation, Formal analysis, Data curation, Writing – original draft, Writing – review & editing. SS: Software, Writing – review & editing, Visualization. KA: Software, Writing – review & editing. MA: Conceptualization, Writing – original draft, Writing – review & editing, Supervision. ZAM: Methodology, Software, Validation, Formal analysis, Data curation, Writing – original draft, Writing – review & editing.
